# Assessment of Multilocus Sequence Analysis (MLSA) for Identification of *Candidatus* Liberibacter Solanacearum from Different Host Plants in Spain

**DOI:** 10.3390/microorganisms8091446

**Published:** 2020-09-21

**Authors:** Ana Ruiz-Padilla, Cristina Redondo, Adrián Asensio, Jerson Garita-Cambronero, Carmen Martínez, Verónica Pérez-Padilla, Raquel Marquínez, Jesús Collar, Eva García-Méndez, Ana Alfaro-Fernández, Carmen Asensio-S.-Manzanera, José Luis Palomo, Felipe Siverio, Leandro De León, Jaime Cubero

**Affiliations:** 1 Instituto Nacional de Investigación y Tecnología Agraria y Alimentaria (INIA), 28040 Madrid, Spain; ana.ruizp@upm.es (A.R.-P.); rcasero@inia.es (C.R.); Ecran99-0@hotmail.com (A.A.); garcamje@itacyl.es (J.G.-C.); veronica.perez@anove.es (V.P.-P.); ldeleon@anove.es (L.D.L.); 2 Instituto Tecnológico Agrario, de Castilla y León (ITACyL), 47071 Castilla y León, Spain; asesanmr@itacyl.es; 3 Instituto Valenciano de Investigaciones Agrarias (IVIA), 46113 Valencia, Spain; cmartine@ivia.es; 4 Asociación Nacional de Obtentores Vegetales (ANOVE), 28014 Madrid, Spain; 5 Laboratorio de Analíticas Vegetales NEIKER_BRTA, 01192 Arkaute, Spain; rmarquinez@neiker.eus; 6 Departamento de Fitopatología, Laboratorio Agrario y Fitopatológico, 15318 Abegondo, Spain; jesus.collar.urquijo@xunta.gal; 7 Centro de Investigación y Formación Agrarias (CIFA), 39600 Muriedas, Spain; garcia_emar@cantabria.es; 8 Instituto Agroforestal Mediterráneo, Universitat Politécnica de València, 46022 Valencia, Spain; analfer1@etsia.upv.es; 9 Centro Regional de Diagnóstico, Junta Castilla y León, 37340 Aldearrubia, Spain; PalGomJo@jcyl.es; 10 Instituto Canario de Investigaciones Agrarias (ICIA), San Cristóbal de La Laguna, 38330 Tenerife, Spain; fsiverio@icia.es

**Keywords:** *Liberibacter*, zebra chip, MLSA, potato, carrot, parsnip, celery, citrus, HLB

## Abstract

*Liberibacter* is a bacterial group causing different diseases and disorders in plants. Among liberibacters, *Candidatus* Liberibacter solanaceraum (CLso) produces disorders in several species mainly within Apiaceae and Solanaceae families. CLso isolates are usually grouped in defined haplotypes according to single nucleotide polymorphisms in genes associated with ribosomal elements. In order to characterize more precisely isolates of CLso identified in potato in Spain, a Multilocus Sequence Analysis (MLSA) was applied. This methodology was validated by a complete analysis of ten housekeeping genes that showed an absence of positive selection and a nearly neutral mechanism for their evolution. Most of the analysis performed with single housekeeping genes, as well as MLSA, grouped together isolates of CLso detected in potato crops in Spain within the haplotype E, undistinguishable from those infecting carrots, parsnips or celery. Moreover, the information from these housekeeping genes was used to estimate the evolutionary divergence among the different CLso by using the concatenated sequences of the genes assayed. Data obtained on the divergence among CLso haplotypes support the hypothesis of evolutionary events connected with different hosts, in different geographic areas, and possibly associated with different vectors. Our results demonstrate the absence in Spain of CLso isolates molecularly classified as haplotypes A and B, traditionally considered causal agents of zebra chip in potato, as well as the uncertain possibility of the present haplotype to produce major disease outbreaks in potato that may depend on many factors that should be further evaluated in future works.

## 1. Introduction

The genus *Liberibacter* is composed by obligate parasites of multiple bacterial species identified in plants worldwide [1]. The species of the genus include the following: *Ca*. L. asiaticus (CLas), *Ca*. L. africanus (CLaf) and *Ca*. L. americanus (CLam), which cause Huanglongbing or HLB in citrus; *Ca*. L. solanacearum (CLso) which affects mainly potato, celery or carrot; *Ca*. L. europeaeus (CLeu) found in Rosaceae plants but considered by some authors as an endophyte rather than a plant pathogen [2] and *Liberibacter crescens* (Lcr) which has been identified on tropical babaco and papaya hybrid and it is the only one able to grow in axenic culture [1,3]. Besides, a new *Candidatus* Liberibacter species, named as *Candidatus* Liberibacter brunswickensis, was detected in a native Australian eggplant psyllid and was not associated to any plant disease [4].

CLso has been detected in America, New Zealand, Europe and the Mediterranean basin associated with damages in economically important crops of the Solanaceae and Apiaceae families [5]. So far, nine haplotypes have been identified for CLso based on the analysis of single nucleotide polymorphisms (SNPs) across partial sequences of 16S rRNA, intergenic spacer region (ISR) and *rplJ* and *rplL* genes. Haplotypes A, B and F were associated with zebra chip disease in potato, meanwhile, C, D and E have been described to cause habitually vegetative disorders in apiaceous plants such as carrot, celery and parsnip, with E also being recently detected in potato in Spain [6]; finally, there was haplotype U, which was identified in nettle plants [7]. In 2019, two more haplotypes were identified, haplotype G in *Solanum umbelliferum*, and haplotype H in Apiaceae and Polygonaceae family plants [8,9]. Each haplotype appears to be found in particular geographical areas and is transmitted by different vectors, with *Bactericera cockerelli* being the vector psyllid for A, B and F haplotypes, *Bactericera trigonica* for haplotypes D and E, *Trioza apicalis* and *Trioza anthrisci* for haplotype C and *Trioza urticae* for haplotype U [5,8]. So far, there is no available information regarding transmission of haplotypes G and H by psyllids.

Characterization and identification of *Liberibacter* types have been based on the gene of the 16S rRNA and the β operon which codes for different proteins of the large ribosomal subunit and subunits B and C of the RNA polymerase [10,11]. However, analysis of single genes may not be totally able to reproduce precise phylogenetic relationships among liberibacters which is why multiple gene-based phylogenetic approaches, such as Multilocus Sequencing Analysis (MLSA) or Multilocus Sequencing Types (MLST), have been applied in the last few years [10,12]. MLSA is based in nucleotide sequencing of fragments from protein-coding genes that evolve at a slow and constant rate and usually shows a higher resolution as compared with ribosomal gene sequence [13]. Nevertheless, MLSA may differ significantly according to the type and number of genes selected [11]. Therefore, this approach needs to be carefully designed and validated to determine its phylogenetic accuracy and its degree of congruence compared with other methodologies or biological characteristics of the bacteria. 

Herein, a comprehensive study was undertaken to evaluate the phylogenetic relationship of all CLso haplotypes previously identified by ribosomal gene sequences in Spain and situate them within the *Liberibacter* context, including information from other species of the genus that produce plant diseases. For that purpose, we have used an MLSA approach based on genes previously utilized in MLST analysis [12] that were selected and concatenated in different combinations. In summary, the aim of the present study was to refine the understanding of the phylogenetic relationships of *Liberibacter* species and help to clarify those found in Spain in different host plants according to a precise methodology.

## 2. Materials and Methods 

### 2.1. Samples Analysed Throughout the Work

The samples analysed in this work are described in Table 1. DNA samples were extracted from carrot (*Daucus carota*), parsnip (*Pastinaca sativa*), potato (*Solanum tuberosum*), tomato (*Solanum lycopersicum*), and pepper (*Capsicum annuum*) from Spain and Mexico (referred in the table as ‘This work’). DNAs for A, B, C and D haplotypes, provided by different research groups, were also used as controls. Finally, data from several isolates were obtained from databases and previous works [5,7,8,9,14,15,16,17,18,19,20,21].

### 2.2. DNA Extraction and Q-PCR Analysis for Detection of CLso

Detection of CLso was conducted by quantitative real-time PCR (q-PCR) on total DNA extracted from plant leaves (carrot), tubers (potato) or true seeds samples (carrot, parsnip, tomato and pepper). For plant leaves, roots and tuber samples, approximately 0.5 g of material were used for DNA extraction. When seed samples were analysed, a pool of 100 seeds was used. The samples were ground with a steel bead for 1 min at 30 rpm using the homogenizer Retsch Mixer Mill MM-400 in 10 mL of phosphate-buffer saline (pH 7.4) supplemented with Tween 20 (0.02%). Then, 1 mL from each homogenized sample was centrifuged at 4 °C for 1 min at a low speed (1000× *g*) to remove the coarse particles. The supernatant was then centrifuged at 4 °C for 7 min at 10,000× *g* to concentrate the bacteria and the pellet used for total DNA extraction by using the DNeasy Plant Mini Kit (Qiagen, Valencia, CA, USA) following the manufacturer’s instructions.

For qPCR analysis, two different combinations of specific primer and probes (Table 2) were used for the detection of CLso [22,23]. PCRs were conducted in a 7500 Fast Real-Time PCR System (Applied Biosystems, Foster City, CA, USA) under the following conditions: an initial cycle of 10 min at 95 °C for initial denaturation followed by 40 cycles of 15s at 95 °C and 1 min at 60 °C. The reaction mix (15 µL) consisted of 7.5 µL of 2 x SensiFAST™ Probe Lo-ROX mix (Bioline Reagents Ltd., London, UK), 0.5 µM of each forward and reverse primers, 0.1 µM for the probe, and 3 µL of extracted total DNA as template. Samples of seeds, potato tubers or carrot plant leaves free of CLso or in the presence of the bacteria were used in each extraction as negative or positive controls, respectively. The efficiency of the DNA extractions was determined by qPCR on total DNA extracts by amplification of the endogenous cytochrome oxidase gene (COX) [24].

### 2.3. Haplotype Identification and Characterization

Haplotypes from samples shown in Table 1 were identified firstly by analysis of 50S ribosomal subunit proteins L10/L12 genes (*rplJ/rplL*) using primers CL514F/R (Table 3) and a protocol previously described [25], and secondly by MLSA.

For MLSA, different primer pairs, determined as useful in MLST studies conducted on Liberibacter [12], were used for PCR amplification of partial sequences of the housekeeping genes *adK*, *dnaG*, *fumC*, *grpE*, *icdA*, *metG*, *mutS*, *purA*, *recA,* and *gyrB* (Table 3). DNAs from samples described in Table 1 were used as targets in PCRs performed as described before for all genes [12] except for *gyrB*. For *gyrB*, PCR amplification was performed in 50 μL volume containing 0.5 μM primers JG-gyrB1 and JG-gyrB2, 0.2 mM dNTPs, and 2 U of Taq polymerase (Promega). The amplification reaction conditions consisted of 94 °C for 1 min, 55 °C for 1 min, and 72 °C for 2 min for 40 cycles, plus an initial step of 94 °C for 5 min and a final step of 72 °C for 10 min. PCR products were visualized in 2% agarose gel containing Midori Green nucleic acid gel staining solution (Nippon GeneticsEurope, Dueren, Germany) and purified with the Wizard SV Gel and PCR Clean-up System Kit (Promega Corporation, Madison, WI, USA). PCR products were sequenced at STABVIDA (Lisbon, Portugal).

Nucleotide sequences were deposited in GenBank. Accession numbers for the partial sequences of the genes used in this study are MT847239 to MT847243 and MT864604 to MT864672.

### 2.4. Phylogenetic Analysis and Molecular Dating of Candidatus Liberibacter Isolates

Sequences were edited and aligned using BioEdit Sequence Alignment Editor [26]. Additionally, sequences from 50S or the housekeeping genes from different *Liberibacter* isolates obtained from the National Center for Biotechnology Information database (NCBI) (http://www.ncbi.nlm.nih.gov) were included in the analysis. Phylogenetic analyses from individual or concatenated genes were conducted in MEGA X [27] using maximum likelihood analysis (ML) and the Tamura-Nei model [28]. A total of 1000 bootstrap re-samplings, were generated in each analysis.

Molecular dating of *Liberibacter* species was estimated based on liberibacters divergence calculated with MEGA X [27] as above and calibrating the molecular clock using the time of divergence between CLas and CLaf, which was previously estimated as 147 million years (Myr) [10].

### 2.5. Descriptive Analysis of the Sequences

The number of polymorphic sites, mutations, nucleotide diversity, average nucleotide differences and Tajima’s test were calculated separately from individual genes or concatenated sequences using DnaSP version 6.12.03 [29].

## 3. Results

### 3.1. Detection of CLso from Different Samples

CLso was detected in samples of potato tubers or seeds from different plant species using two different primer-probe specific assays for qPCR. To rule out the presence of inhibitors in qPCR reactions, often present in difficult samples such as tubers and seeds, detection of the COX gene was used in each sample as an internal amplification control. Only samples that tested positive using the two specific qPCR assays for CLso were used for the subsequent sequence analysis.

### 3.2. 50S Ribosomal Subunit Proteins L10/L12 (rplJ/rplL) Genes Sequence Analysis

Sequence analysis of the partial 50S ribosomal proteins L10/L12 genes (*rplJ/rplL*) confirmed that all 39 isolates from either the database or those obtained in this work belonged to *Liberibacter* species. Some of these sequences included those found in potato samples in Spain. The analysis showed similarities in *rplJ/rplL* sequences ranging from 62.69 to 99.76 (*p*-distance in %). The mean similarity level (±SE) on this gene among liberibacters was 91.09 ± 0.52 with a maximum of 150 nucleotide differences over a sequence of 453 nt positions. Within CLso isolates, a mean similarity of 98.48 ± 0.33 and an average nucleotide difference of 6.54 ± 1.42 were elucidated. A mean similarity of 76.52 ± 1.40 and an average nucleotide difference of 97.33 ± 5.93 were found among HLB liberibacters and no differences were shown between the two Lcr isolates studied. Moreover, the mean similarity between CLso and HLB or Lcr isolates was 76.15 ± 1.53 (100.61 ± 6.38 nt) and 66.09 ± 2.21 (145.36 ± 9.23 nt), respectively; finally, that between HLB and Lcr isolates was 65.77 ± 1.79 (144.75 ± 7.28 nt).

**Figure 1:**
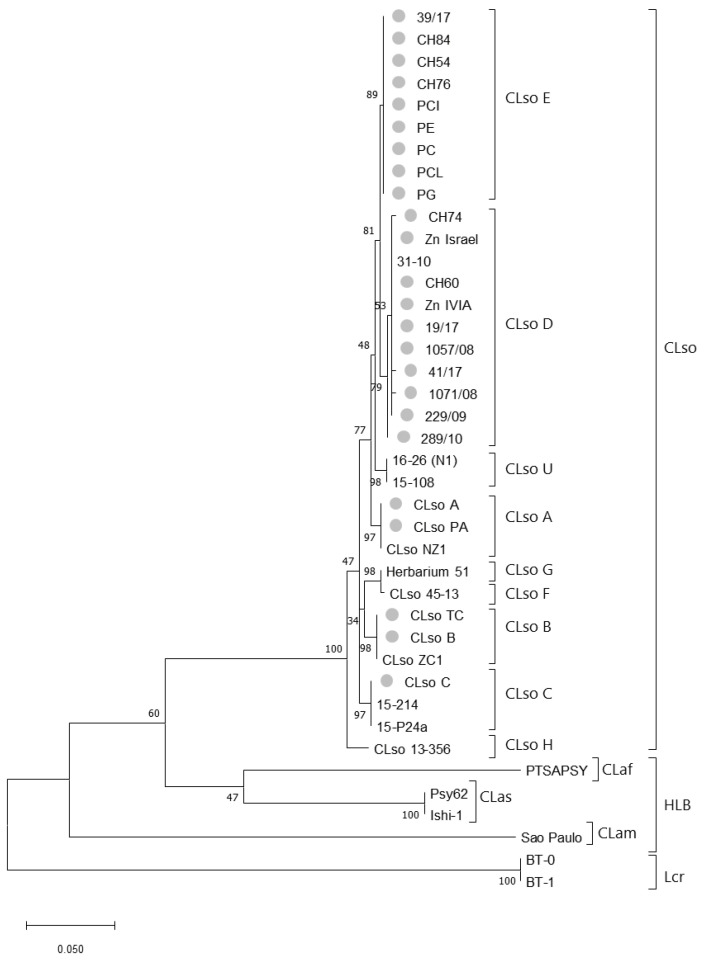
Phylogenetic tree of *Liberibacter* strains based on partial sequences of the 50S ribosomal proteins L10/L12 genes (*rplJ/rplL*). The tree was based on nucleotide sequences from 39 isolates and 453 nt positions. Analysis was performed using the Maximum Likelihood method and the Tamura-Nei model [28]. Bootstrap values (1000 replications) are shown at the branch points. Grey circles indicate those samples sequenced in this work. Phylogenetic analyses were conducted in MEGA X [27].

The phylogenetic tree constructed using the Maximum Likelihood method and the Tamura-Nei model, inferred different phylogroups corresponding to the different types of *Liberibacter* species or haplotypes. CLso samples were clustered together and separated from HLB species or Lcr. Besides, clear different clusters were associated within the CLso haplotypes, but with close connections between D and E or F and G haplotypes. All haplotypes detected in Spain were included in D and E groups, even those isolates detected in potato tubers that differentiated from A, B, and F haplotypes causing zebra chip or infecting potato, tomato, and pepper in other areas of the word (Figure 1).

To study in greater depth the relationship among the different CLso haplotypes, the mean similarity among these different groups was evaluated (Table 4). The analysis showed similarities among different isolates ranging from 96.71 ± 0.85 to 99.77 ± 0.23. The lowest differences were shown between D and E, and F and G haplotypes, and the highest between H and the rest of the haplotypes, as shown also in the phylogenetic tree (Figure 1). Similarities between haplotype from potato in Spain and haplotypes A and B causing zebra chip in America were 98.84 ± 0.52 and 97.68 ± 0.01, respectively.

Compared with the CLso group, higher differences were observed among the *Liberibacter* species causing HLB, showing mean similarities between CLam and CLas, CLam and CLaf, or CLas and CLaf of 66.58 ± 2.31, 67.65 ± 2.26 and 78.60 ± 2.01, respectively.

### 3.3. MLSA Sequence Analysis

The MLSA study was conducted based on the sequence analysis of housekeeping genes *adk*, *gyrB*, *dnaG*, *fumC, grpE*, *icdA*, *metG*, *mutS*, *purA,* and *recA.*

**Figure 2:**
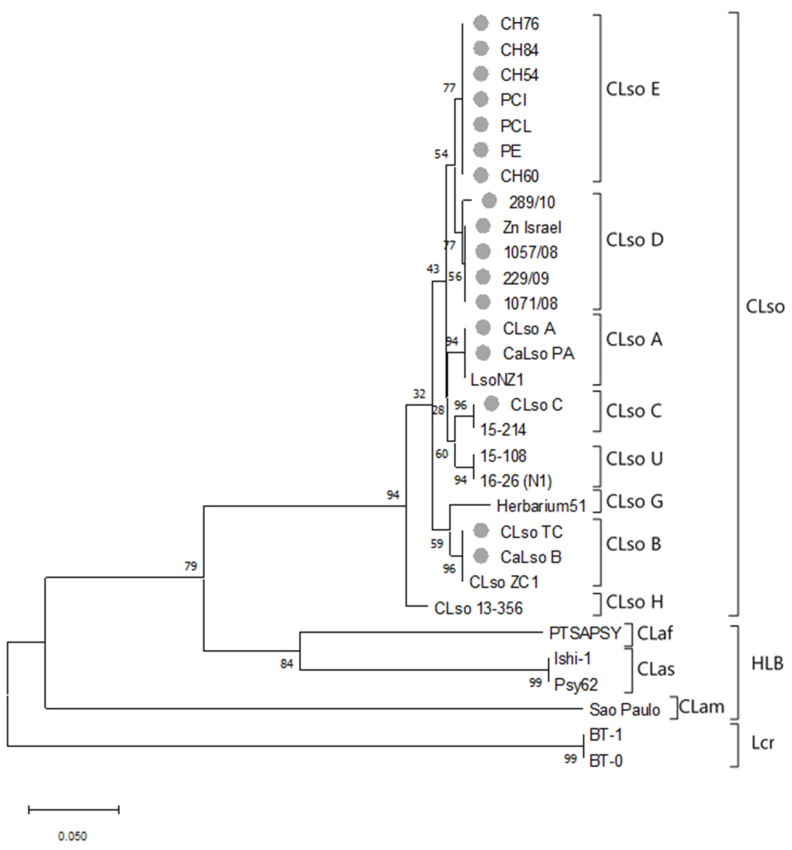
Example of phylogenetic tree of representative *Liberibacter* strains based on partial sequence of adenosine kinase gene (*adk*). The tree was based on nucleotide sequences from 30 isolates and 208 nt positions. Analysis was performed using the Maximum Likelihood method and the Tamura-Nei model [28]. Bootstrap values (1000 replications) are shown at the branch points. Grey circles indicate those sequenced in this work. Phylogenetic analyses were conducted in MEGA X [27].

Evolutionary information was calculated for each housekeeping gene either in CLso alone or including *Liberibacter* species causing disease in citrus, and *L. crescens,* which is known to be taxonomically far apart from CLso or HLB isolates. No significant sequence variations were found among isolates belonging to the same *Liberibacter* species or haplotype as shown in an example in Figure 2; thus, a representative sequence of each group was selected for further analysis.

To obtain evolutionary information of each gene, different parameters were considered. Data regarding *Liberibacter* and CLso evolution are shown in Table 5. Negative Tajima’s D values resulted as a consequence of fewer haplotypes than segregating sites. This indicates low-frequency polymorphisms and suggests scenarios of a population size expansion after a bottleneck or a selective sweep and/or purifying selection. Higher differences were found between haplotypes and segregation sites when all liberibacters were included in the analysis compared with that shown when just CLso was studied, reinforcing the idea of an absence of positive selection but a nearly neutral mechanism for the analysed housekeeping gene evolution [30].

**Figure 3:**
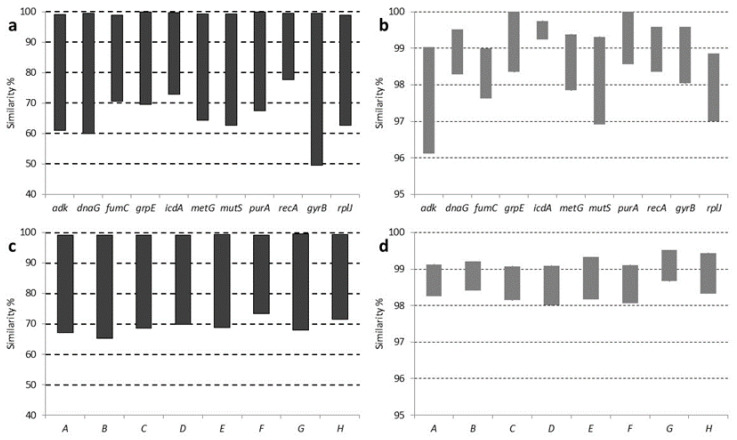
Taxonomic resolution based on the number of base differences per site (*p*-distance) between sequences from the *Liberibacter* (**a**,**c**) and CLso (**b**,**d**) phylogroups using individual genes (**a**,**b**) or multilocus sequence analysis (MLSA) approaches (**c**,**d**) by different concatenated gene combinations: *adk-mutS-icdA* (A), *adk-dnaG-icdA* (B), *adk-dnaG-recA* (C)*, adk-mutS-recA* (D,), *adk-icdA* (E), *adk-recA* (F), *mutS-icdA* (G), *mutS-recA* (H). The evolutionary history was inferred using the Maximum Likelihood method and the Tamura-Nei model [28]. Bootstrap values (1000 replications). Analysis was conducted in MEGA X [27].

In order to select those genes to be used in the MLSA approach, a precise study about inter-*Liberibacter* and intra-CLso similarity was performed. The inter-*Liberibacter* phylogroup gene sequence interval similarities were 99.03–60.98, 99.51–60.00, 98.99–70.51, 100.00–69.61, 99.75–72.91, 99.38–64.31, 99.31–62.70, 100.00–67.38, 99.57–77.68, and 99.58–49.56 for *adK*, *dnaG*, *fumC*, *grpE*, *icdA*, *metG*, *mutS*, *purA*, *recA,* and *gyrB*, respectively and 98.85–62.60 for *rplJ/rplL*. The intra-CLso phylogroup gene sequence similarities were 99.03–96.12, 99.51–98.29, 98.99–97.63, 100.00–98.35, 99.75–99.24, 99.38–97.85, 99.31–96.92, 100.00–98.57, 99.57–98.71 and 99.58–98.04 for *adK*, *dnaG*, *fumC*, *grpE*, *icdA*, *metG*, *mutS*, *purA*, *recA* and *gyrB*, respectively and 98.85–97.00 for *rplJ*/*rplL* (Figure 3a,b).

**Figure 4:**
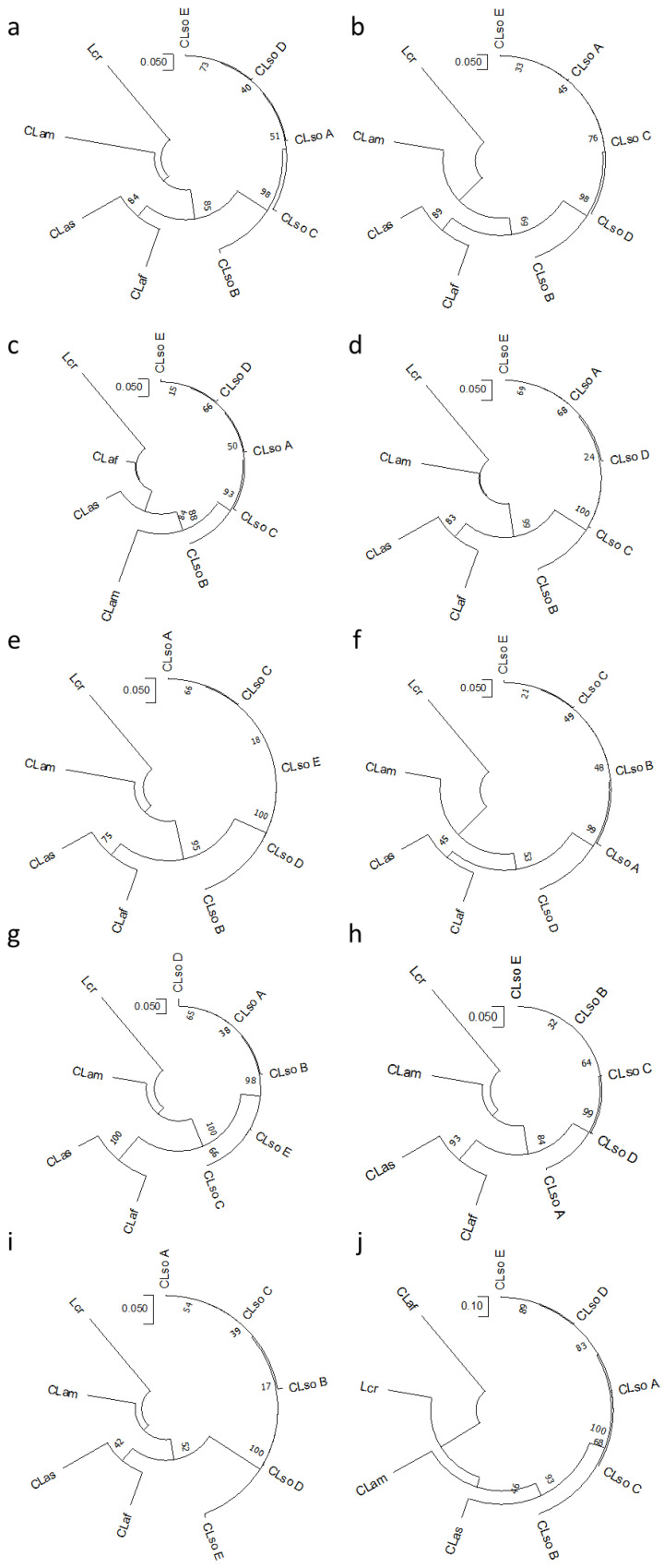
Phylogenetic tree of representative isolates of CLso, CLas, CLaf, and Lcr based on partial sequence of *adk* (**a**), *dnaG* (**b**), *fumC* (**c**), *grpE* (**d**), *icdA* (**e**), *metG* (**f**), *mutS* (**g**), *purA* (**h**), *recA* (**i**) and *gyrB* (**j**). Analysis was performed by using the Maximum Likelihood method and the Tamura-Nei model [28]. Bootstrap values (1000 replications) are shown at the branch points.

In addition, a separated phylogenetic analysis was performed for each gene using sequences for CLso haplotypes A, B, C, D, and E, obtained in this work, but also including information for other liberibacters available in databases. The phylogenetic trees revealed, most of the time, similar topologies, although some variations were found among the different genes analysed. Every CLso isolate was always included in the same phylogroup and separated from isolates that cause HLB in citrus species or Lcr (Figure 4). Moreover, different species of *Liberibacter*, causing HLB in citrus were clearly separated; meanwhile, cluster division within CLso isolates was not as noticeable. 

Overall, in comparison with the *rplJ/rplL* genes tree, phylogenetic trees predicted from individual housekeeping gene sequences presented congruent phylogroups. However, in some cases, the clustering was poorly resolved and weakly supported by bootstrap values and within CLso, some kind of disagreement among the trees was shown. In the case of *gyrB* gene, a strong discrepancy was shown in the phylogenic separation between HLB isolates and Lcr as compared with that obtained by the other genes (Figure 4).

To overcome the possible evolutionary differences of the genes and the putative poor resolution of the single-gene analysis, an MLSA approach based on concatenated sequence analysis was performed. Sequences from the ten housekeeping genes previously studied were used in the analysis, and those showing more suitable features based on single-gene analysis were selected and combined for MLSA analysis.

Similarl to previous analysis, the phylogenetic tree inferred different phylogroups corresponding to *Liberibacter* species or haplotypes. CLso isolates were clustered together and separated from HLB species or Lcr. Different clusters were associated with different CLso haplotypes with higher bootstrap values that supported those obtained by *rplJ/rplL*.

**Figure 5:**
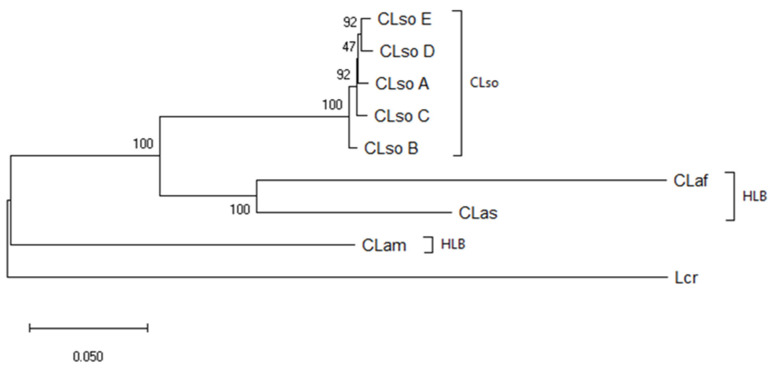
Phylogenetic tree of representative isolates of CLso, CLas, CLam, CLaf and Lcr based on concatenated sequences obtained from *adk*, *dnaG*, *fumC*, *grpE*, *icdA*, *metG*, *mutS*, *purA*, *recA* and *gyrB*. The evolutionary history was inferred using the Maximum Likelihood method and the Tamura-Nei model [28]. Bootstrap values (1000 replications) are shown at the branch points. A total of 3967 nt positions in the final dataset were analysed in MEGA X [27].

Haplotypes D and E were apart from haplotypes C and B and less separated from A (Figure 5). As a whole, the analysis of housekeeping genes provides a robust haplotype delineation equivalent to the ribosomal analysis but supported by different genetic features.

### 3.4. Concatenated Sequence Analysis

In order to determine which and how many genes were needed to be used in the MLSA analysis to achieve a robust delineation of the different *Liberibacter* species and haplotypes, different gene combinations were evaluated. Gene selection was performed according to the similarity range previously determined for single genes (Figure 3 and Figure 4). *adk*, *mutS*, *icdA,* and *recA* were selected for MLSA being those that showed the widest and narrowest ranges of similarity within liberibacters or CLso isolates. 

The inter-*Liberibacter* phylogroup sequence interval similarities were 99.13–67.15, 99.21–65.25, 99.08–68.55, 99.09–69.82, 99.33–68.83, 99.11–73.32, 99.52–67.96, and 99.44–71.40 for gene combinations: *adk-mutS-icdA*, *adk-dnaG-icdA*, *adk-dnaG-recA, adk-mutS-recA*, *adk-icdA*, *adk-recA*, *mutS-icdA* and *mutS-recA,* respectively. The intra-CLso phylogroup sequence interval similarities were 99.13–98.26, 99.21–98.42, 99.08–98.15, 99.09–98.01, 99.33–98.17, 99.11–98.07, 99.52–98.67 and 99.44–98.33 for the following gene combinations: *adk-mutS-icdA*, *adk-dnaG-icdA*, *adk-dnaG-recA, adk-mutS-recA*, *adk-icdA*, *adk-recA*, *mutS-icdA* and *mutS-recA*, respectively (Figure 3c,d).

**Figure 6:**
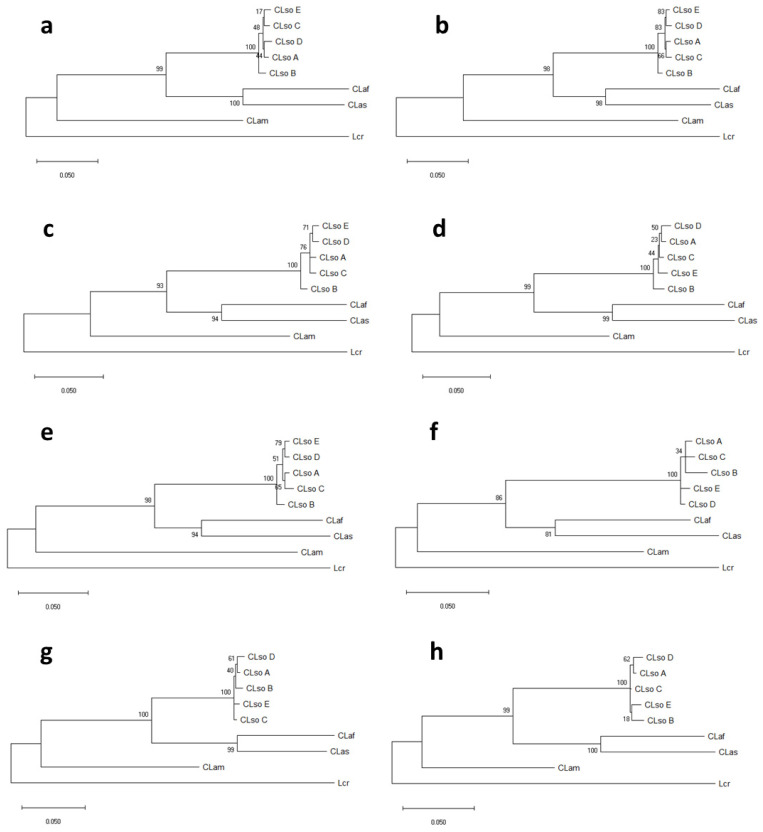
Phylogenetic trees of CLso, CLas, CLaf and Lcr based on concatenated sequences obtained from *adk-mutS-icdA* (**a**), *adk-dnaG-icdA* (**b**), *adk-dnaG-recA* (**c**)*, adk-mutS-recA* (**d**), *adk-icdA* (**e**), *adk-recA* (**f**), *mutS-icdA* (**g**), and *mutS-recA* (**h**). The evolutionary history was inferred using the Maximum Likelihood method and the Tamura-Nei model [28]. Bootstrap values (1000 replications) are shown at the branch points. A total of 1037 (*adk-mutS-icdA*), 1012 (*adk-dnaG-icdA*), 1083 (*adk-dnaG-recA*), 1108 (*adk-mutS-recA*), 602 (*adk-icdA*), 673 (*adk-recA*), 830 (*mutS-icdA*), and 901 (*mutS-recA*) positions in the final dataset were analysed in MEGA X [27].

The topology of the eight trees inferred from the different concatenated gene combinations was found to be quite similar among them and comparable to the one based on the ribosomal *rplJ/rplL* genes (Figure 6). CLso haplotypes defined a single cluster clearly separated from those liberibacters causing HLB or Lcr. However, a more detailed analysis allowed to detect some clustering differences between CLso haplotypes D and E, detected in Spain, and the other haplotypes analysed (Figure 6). As for individual genes, the evolutionary information from each gene combination was studied. Table 6 shows the average pairwise nucleotide diversity and the difference per site and sequence, the number of mutations and segregation sites, as well as Tajima’s *D* values.

All the Tajima’s *D* values were negative in the concatenated sequences, confirming the data obtained previously with individual genes that is lower average heterozygosity and population expansion after a recent bottleneck event linked to swept genes. The gene combination showed slight differences providing the highest diversity in the *Liberibacter* context with the concatenated genes *adk-mutS-icdA* and for CLso with the combination *adk-icdA.*

### 3.5. Molecular Dating on Liberibacter Species

**Figure 7:**
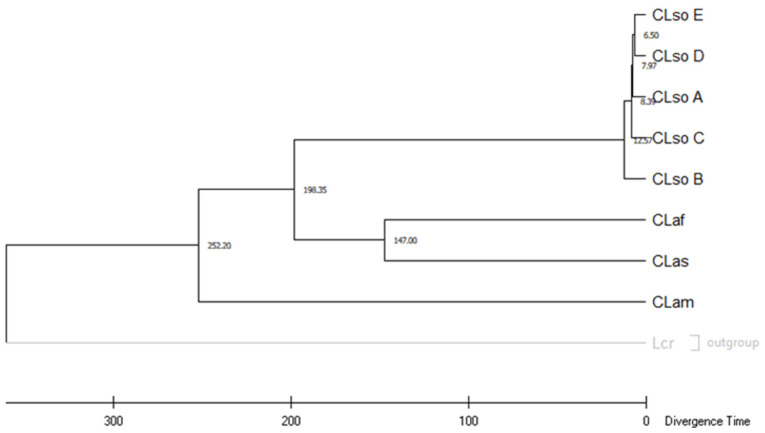
Timetree inferred by applying the RelTime method [31,32] to the user-supplied phylogenetic tree whose branch lengths were calculated using the Maximum Likelihood (ML) method and the Tamura-Nei substitution model [28]. The time tree was computed using one calibration constraint (147 Myr between CLas/CLaf). This analysis involved nine nucleotide sequences and BT1 of *L. crescens* was used as outgroup. There were a total of 3933 positions in the final dataset. Evolutionary analyses were conducted in MEGA X [27].

To infer the evolutionary dynamic of *Liberibacter* species a time tree was constructed based on concatenated sequence analysis of the all house-keeping genes evaluated above. Equal evolutionary rates in all lineages were assumed and the clock was calibrated to convert distance to time using the time of divergence between CLas and CLaf estimated previously as 147 Myr [10]. Using this parameter, the time of divergence between CLso E and CLso D was 6.50 Myr, between CLso D or CLso E and CLso A was 7.97 Myr, and between these three and CLso C was 8.39 Myr. Finally, the longest time was found between CLso B and the rest and was estimated at 12.57 Myr. The distance obtained between CLam and CLas was 252.20 Myr, in similar range to that obtained previously [10] (Figure 7).

## 4. Discussion

Many *Liberibacter* species have been described as pathogens and associated as the causal agents of serious diseases such as HLB or zebra chip. However, in the last few years, other bacteria belonging to this group were described as non-pathogenic or just causing minor, unclear, or indirect damages in plants [1,2,3,4]. Among the *Liberibacter* species, different CLso haplotypes, generally linked to different geographic regions, have been described to cause disease in a variety of economically important crops [1]. This situation may resemble that described for the different HLB liberibacter types, considered as the result of an evolutionary divergence resulting in species diversity according to the origin [3].

Haplotype determination in CLso has been conventionally based on single nucleotide polymorphisms in genes associated with ribosomal elements. However, analysis of single genes may not reflect general phylogenetic relationships in prokaryotes. To overcome this limitation, multiple gene-based phylogeny approaches, such as MLSA or MLST, have been introduced for genomic analysis [11,12,13]. MLSA is based on nucleotide sequencing of internal fragments of protein-coding genes and subsequent phylogenetic analysis [13]. This technique generally shows higher resolution compared with ribosomal RNA sequencing, as protein-coding genes evolve at a slow and constant rate, providing better resolution power [33]. In our study, the MLSA phylogeny roughly confirmed the ribosomal gene-based grouping previously suggested [7,8,9,10,12,14,34], but revealed more precise and robust information about the genetic relationships of different *Liberibacter* haplotypes and species. Phylogenetic trees predicted from individual housekeeping genes in CLso showed some differences within the species. The different location of the haplotypes in the single-gene tree analysis could indicate a different evolutionary process, such as recombinant events, horizontal gene transfer, or intragenomic rearrangements [33]. These slight differences among the housekeeping genes in CLso haplotypes reveal a short genetic distance among them, similar to that shown when ribosomal sequences were analysed. The discrepancies observed in tree topologies from individual genes reinforced the need to approach this study using multilocus analysis including multiple independent genes to achieve more accurate group phylogeny.

Our MLSA analysis was based on the sequence analysis of housekeeping genes used in a previous MLST study [12]. A total of ten genes were analysed and the selected combinations were approached based on two main criteria: (i) the ease of amplifying the target sequence from all the isolates, and (ii) the suitability of each housekeeping-gene chosen from an evolutionary perspective. A non-cultivable microorganism such as CLso requires PCR amplification of the target sequence from DNA extracted from plant material. Herein, after appropriate DNA extraction, all the genes selected were relatively easily amplified from infected plant material. In addition, the analysis of short sequences from PCR products was acceptable for the phylogenetic studies, and no cross reactions with other bacterial DNA were obtained after sequencing. Moreover, the low frequency of polymorphisms and the absence of positive selection of the studied genes suggest a nearly neutral mechanism for their evolution. Thus, all the selected genes showed similar features and, therefore, could be applied in an MLSA approach, as occurred in other bacterial models [35].

A gene concatenation has been defined to provide better results than a single gene analysis because it predicts intraspecific relationships more accurately [36]. Here, the concatenated genes were also chosen according to their individual taxonomic resolution inter-*Liberibacter* and intra-CLso. Genes presenting widest and narrowest similarities among the isolates were selected to avoid over and underestimation of the genetic distance among the liberibacters studied, and then assembled and evaluated as a single sequence. Our results demonstrated that the combinations of two or three key genes may be sufficient to reveal the precise phylogenetic position of the different liberibacters. In many cases, the information provided by these few key genes was similar to that obtained after analysis of the ten concatenated genes. The consistency in the classification of *Liberibacter* isolates by MLSA was given by the bootstrap analysis of the phylogenetic trees as well as by the evolutionary features of the concatenated genes.

Classification of the *Liberibacter* isolates is not just an academic or taxonomic issue. Many of the *Liberibacter* species are considered important pathogens in agriculture, and their classification can seriously affect many crops, the international trade, and the associated sectoral economy. Currently, new species of *Liberibacter* are being identified and it is likely that other new ones, associated or not with plant diseases, will be detected in plants or insect vectors in the near future [37]. By then, it will be essential to determine if these new isolates are plant pathogens as well as their relationship with the isolates or variants already characterized. This can be achieved with appropriate phylogenetic studies and through the use of different characters that average the evolution of different genetic features. Indeed, our work aimed to determine the phylogenetic position of those haplotypes of liberibacters detected in Spain commonly associated with apiaceous crops but able to infect potato tubers. An initial ribosomal sequence analysis grouped the CLso isolates detected in potato crops within the haplotype E, undistinguishable from those infecting carrots, parsnips, or celery. Most of the analysis performed with single genes grouped E and D haplotypes at a certain distance from A, B, and C.

It should be noted that the characterization of the haplotype is important from a molecular tool perspective, but it may not be related to bacterial pathogenicity and it is not addressed to directly identify characters involved in virulence or host range. Virulence or host range markers should be determined after genomic analysis from the available genomes or after whole genomic sequencing of isolates as they emerge [38]. So far, an MLSA approach provides valuable information regarding CLso relationship between their suggested pathogenicity in certain plant hosts and their evolution.

Taking advantage of the previously published data for HLB in citrus [10] and using the information obtained from the analysed housekeeping genes, a molecular dating for CLso was inferred. To estimate the time of splitting among the CLso isolates, the evolutionary divergence among the different isolated was calculated using the concatenated sequence of the ten genes evaluated. The clock was calibrated using the time of divergence between African and Asian liberibacters which was estimated at 147 Myr ago [10]. Shorter distances were obtained within the CLso haplotypes, with the shortest being between haplotypes D and E, and the longest between B and the rest of the haplotypes with a distance of more than double that estimated between D and E. These data on the divergence among CLso haplotypes support the hypothesis that the evolution of the different isolates maybe associated with different hosts, in different geographic areas and, probably connected to different vectors [39]. Why are some CLso haplotypes more prevalent in a particular host for example A and B for potato in America versus D and E for carrots, parsnip and celery in Europe? Are the genetic differences found in CLso haplotypes sufficient to play a key role in differentiating the host range or are other factors involved? In Spain, only haplotypes D and E were identified, both efficiently transmitted by the psyllid *B. trigonica* and very closely related to apiaceous plants. However, haplotype E has also been detected sporadically in potato growing areas, mainly in tubers and a few records in plants. Although the detection of haplotype E in potato in Spain is considered a type of incidental event [39], the potato plants displayed the typical symptoms attributed to the presence of haplotypes A and B in regions where they are present. Thus, the minor affectation in potato in this region could be more related to the prevalence of the vector *B. trigonica*, a psyllid that has shown its preferences into settle, feed, and oviposit on apiaceous plants, than with genomic variations observed in different haplotypes of CLso [39,40]. Still, another possibility is the existence of a different host range based on genomic variations between haplotypes A or B and D or E, but, so far, such association has not been identified between haplotypes [15,16,41,42]. New genomic and proteomic approaches are desirable to understand the infection processes and elucidate the factors that rule the host range and virulence of these bacteria. In Spain, these studies are addressed to evaluate the possible evolution from haplotype D to E and their transmission by vectors, which may be determining factors that can play a key role in the infection of different hosts [43].

What is clear is the absence in Spain of CLso haplotypes molecularly classified as producing the typical zebra chip disease. However, a definitive answer to the possibility that current haplotypes cause a major outbreak in potato crops will depend on ongoing studies on the transmissibility of haplotype E by an efficient vector, or by a more in-depth genomic analysis to determine differential factors that may govern the host range or virulence of the pathogen.

## Figures and Tables

**Table 1:** *Liberibacter* Isolates Analysed.

Sample	Source	Origin	Type	Reference
39/17 ^1^	Carrot/seeds	Castilla y León/Spain	CLso E	This work
CH84 ^1^	Parsnip/seeds	Valencia/Spain	CLso E	This work
CH54 ^1^	Parsnip/seeds	Valencia/Spain	CLso E	This work
CH76 ^1^	Parsnip/seeds	Valencia/Spain	CLso E	This work
PCI ^1^	Potato/tubers	Canary/Spain	CLso E	This work
PE ^1^	Potato/tubers	Euskadi/Spain	CLso E	This work
PC ^1^	Potato/tubers	Cantabria/Spain	CLso E	This work
PCL ^1^	Potato/tubers	Castilla y León/Spain	CLso E	This work
PG ^1^	Potato/tubers	Galicia/Spain	CLso E	This work
Zn IVIA ^1^	Carrot/seeds	Valencia/Spain	CLso D	This work
19/17 ^1^	Carrot/seeds	Castilla y León/Spain	CLso D	This work
41/17 ^1^	Carrot/seeds	Castilla y León/Spain	CLso D	This work
CH60 ^1^	Parsnip/seeds	Valencia/Spain	CLso D	This work
CH74 ^1^	Parsnip/seeds	Valencia/Spain	CLso D	This work
CLso TC ^1^	Tomato/seeds	Mexico	CLso B	This work
CLso PA ^1^	Pepper/seeds	Mexico	CLso A	This work
CLso C ^2^	Unknown	Unknown	CLso C	IVIA control
CLso B ^2^	Unknown	Unknown	CLso B	IVIA control
CLso A ^2^	Unknown	Unknown	CLso A	IVIA control
Zn Israel ^3^	Carrot/leaf	Israel	CLso D	Volcani C
31–10 ^4^	Carrot/leaf	Canary/Spain	CLso D	[5]
289/10 ^4^	Carrot/leaf	Valencia/Spain	CLso D	[5]
1057/08 ^4^	Carrot/leaf	Valencia/Spain	CLso D	[5]
1071/08 ^4^	Carrot/leaf	Valencia/Spain	CLso D	[5]
229/09 ^4^	Celery/leaf	Valencia/Spain	CLso D	[5]
15-214 ^5^	*Trioza anthisci*	Finland	CLso C	[8]
15-P24a ^5^	*Trioza apicalis*	Finland	CLso C	[8]
CLso 13-356 ^5^	Carrot/leaf	Finland	CLso H	[8]
16-26 (N1) ^5^	Nettle plants	Finland	CLso U	[7]
15-108 ^5^	*Trioza urticae*	Finland	CLso U	[7]
Herbarium 51 ^5^	*Solanum umbelliferum*	USA	CLso G	[9]
CLso 45-13 ^5^	Potato/tuber	USA	CLso F	[14]
CLso ZC1 ^5^	*Bactericera cockerelli*	USA	CLso B	[15]
CLso NZ1 ^5^	*Bactericera cockerelli*	New Zealand	CLso A	[16]
PTSAPSY ^5^	*Citrus* sp.	South Africa	CLaf	[17]
São Paulo ^5^	*Citrus* sp.	Brazil	CLam	[18]
Psy62 ^5^	*Citrus* sp.	USA	CLas	[19]
Ishi-1 ^5^	*Citrus* sp.	Japan	CLas	[20]
BT-0 ^5^	Mountain Papaya	Puerto Rico	Lcr	[21]
BT-1 ^5^	Mountain Papaya	Puerto Rico	Lcr	[21]

^1^ DNA obtained in this work, DNA provided by the following: ^2^ Instituto Valenciano de Investigaciones Agrarias (IVIA), (Spain), ^3^ Volcani Center (Israel), and ^4^ Universitat Politécnica de València (UPV), ^5^ Data obtained from public databases.

**Table 2:** Primers and Probes Used for qPCR Reactions to Specifically Detect CLso and the Endogenous Plant Gene, Cytochrome Oxidase (COX) in Samples of Leaves, Roots, Seeds or Potato Tubers.

Oligos	Sequence (5′-3′)	Target	Ref.
CaLsppF	GCAGGCCTAACACATGCAAGT	16S rRNA	[22]
CaLsppR	GCACACGTTTCCATGCGTTAT		
CaLspl ^1^	FAM-AGCGCTTATTTTTAATAGGAGCGGCAGACG-TAMRA		
LsoF	GTCGAGCGCTTATTTTTAATAGGA	16S rRNA	[23]
HLBr	GCGTTATCCCGTAGAAAAAGGTAG		
HLBp ^1^	FAM-AGACGGGTGAGTAACGCG-BHQ-1		
COXf	GTATGCCACGTCGCATTCCAGA	Cytochrome oxidase (COX)	[24]
COXr	GCCAAAACTGCTAAGGGCATTC		
COXp ^1^	TET-ATCCAGATGCTTACGCTGG-BHQ-2		

^1^ q-PCR probes.

**Table 3:** Primers used in PCRs for haplotype characterization.

Oligos	Sequence (5′-3′)	Target	Ref.
CL514F	CTCTAAGATTTCGGTTGGTT	50S proteins L10/L12 genes (*rplJ/rplL*)	[25]
CL514R	TATATCTATCGTTGCACCAG		
ADK	ATGAGAATTATATTTCTAGGCCCTCC	adenylate kinase (*adk*)	[12]
CKC_0526 0	ATCATATTTATCATCTGATCGCACAG		
DnaG	TTGCTATTGACTTTGATTAATCATCC	DNA primase (*dnaG*)	[12]
CKC_05195	CAAAGCCTTCTATTATGGCTTCTTG		
FumC	TTCCTTTAGTCGTCTGGCAAACAGG	fumarate hydratase (*fumC*)	[12]
CKC_050 75	ACTTGTGCAGCGTATCCTGAAAATTC		
GrpE	TAGAAATACCAACTAAAGCGGGGCG	heat shock protein (*grpE*)	[12]
CKC_00585	GGAAATCCCCTAACGGAACCATTCG		
IcdA	TAGAAATACCAACTAAAGCGGGGCG	isocitrate dehydrogenase (*icdA*)	[12]
CKC_043 65	GGAAATCCCCTAACGGAACCATTCG		
MetG	TCGTACGATGATTTTATTCGCACAACGG	methionyl-tRNA synthetase (*metG*)	[12]
CKC_02965	GGATCGTTAGGAATTTTTATTCCCCAATC		
MutS	CCAACAGATTCTAATTATCTCATGG	DNA mismatch repair protein (*mutS*)	[12]
CKC_008 15	TCTAAATTGGAACGAGCGGCGGA		
PurA	TGTAGTTGTGGTCGGCTTACAATGG	adenylosuccinate synthetase (*purA*)	[12]
CKC_003 15	TATCTTCATAAGCTGGGCCAATACC		
RecA	TTGGAAATAACAGATATGCTGGTGCG	recombinase A (*recA*)	[12]
CKC_050 85	AACCACGCTCCTGATTTATCAACGAT		
JG-gyrB1	AACGCTAGCCGTCTTGTGAA	DNA topoisomerase IV sub B (*gyrB*)	This work
JG-gyrB2	TTGCCACGCAAGGGAAGTAT		

**Table 4:** Average Similarity (*p*-distance) Over All Sequence Pairs Between CLso Groups of Haplotypes Based on Partial Sequences of the 50S Ribosomal Proteins L10/L12 Genes (*rplJ/rplL*).

	CLso A	CLso B	CLso C	CLso D	Clso E	CLso F	CLso G	CLso H	CLso U
**CLso A**		0.65 ^1^	0.59	0.58	0.50	0.68	0.64	0.79	0.56
**CLso B**	97.91		0.53	0.77	0.71	0.63	0.58	0.77	0.67
**CLso C**	98.38	98.61		0.68	0.61	0.68	0.62	0.73	0.58
**CLso D**	98.38	97.22	97.69		0.43	0.78	0.73	0.79	0.59
**CLso E**	98.84	97.68	98.15	99.07		0.71	0.67	0.81	0.52
**CLso F**	97.90	98.13	97.91	97.20	97.66		0.23	0.78	0.69
**CLso G**	98.14	98.37	98.15	97.44	97.91	99.77		0.73	0.64
**CLso H**	96.98	97.22	97.47	96.71	96.75	97.22	97.46		0.78
**CLso U**	98.61	97.91	98.38	98.38	98.84	97.90	98.14	96.98	

^1^ Standard error estimate(s) are shown above the diagonal. This analysis involved 39 nucleotide sequences and a total of 453 positions in the final dataset. Phylogenetic analyses were conducted in MEGA X [27]. Grey scale represents the level of sequence similarity from the lowest (brightest) to the highest (darkest).

**Table 5:** Descriptive Analysis of Nucleotide Sequence Data for Each Housekeeping Gene on *Liberibacter* spp and CLso.

Gene	No. of Mutations	No. of Polymorphic Sites	ds ^1^	k ^2^	Tajima’s *D* Value
*rplJ/rplL* ^3^	291	223	0.09040	35.97841	−1.78652
*rplJ/rplL* ^4^	19	19	0.01076	4.63768	−0.32106
*adk* ^3^	173	120	0.22971	46.86111	−1.36942
*adk* ^4^	13	13	0.02621	5.40000	−0.97762
*dnaG* ^3^	286	223	0.19587	80.30556	−1.23317
*dnaG* ^4^	11	11	0.01073	4.40000	−1.19955
*fumC* ^3^	148	114	0.14153	41.75000	−1.20958
*fumC* ^4^	11	11	0.01549	4.60000	−0.95426
*grpE* ^3^	225	167	0.17818	64.50000	−1.14858
*grpE* ^4^	7	7	0.00826	3.00000	−0.74682
*icdA* ^3^	203	161	0.14866	58.72222	−1.11107
*icdA* ^4^	5	5	0.00557	2.40000	−0.56199
*metG* ^3^	194	163	0.16769	54.50000	−1.22856
*metG* ^4^	11	11	0.01354	4.40000	−1.19955
*mutS* ^3^	223	281	0.19543	82.66667	−1.11084
*mutS* ^4^	14	14	0.01389	6.00000	−0.78089
*purA* ^3^	160	125	0.15681	43.75000	−1.33243
*purA* ^4^	5	5	0.00717	2.00000	−1.12397
*recA* ^3^	195	150	0.12953	60.36111	−0.82453
*recA* ^4^	10	10	0.00944	4.40000	−0.59633
*gyrB* ^3^	611	465	0.23816	163.58333	−1.42139
*gyrB* ^4^	18	18	0.01178	8.40000	−0.20459

^1^ Average nucleotide diversity. ^2^ Average nucleotide differences. Analysis performed on ^3^ *Liberibacter* spp or ^4^ CLso. Nucleotide sites analysed: *rplJ/rplL* (453), *adk* (208), *dnaG* (413), *fumC* (302), *grpE* (373), *icdA* (395), *metG* (333), *mutS* (456), *purA* (279), *recA* (466), and *gyrB* (736).

**Table 6:** Descriptive Analysis of Nucleotide Sequence Data for Concatenated Sequences Used in Multilocus Sequence Analysis (MLSA) Approaches.

Gene	No. of Mutations	No. of Polymorphic Sites	ds ^1^	k ^2^	Tajima’s *D* Value
*A* ^3^	657	504	0.18420	187.11111	−1.17949
*A* ^4^	32	32	0.01317	13.60000	−0.85795
*B* ^3^	662	504	0.18423	185.88889	−1.23622
*B* ^4^	29	29	0.01187	12.00000	−1.03047
*C* ^3^	654	493	0.17364	187.52778	−1.15191
*C* ^4^	34	34	0.01312	14.20000	−0.97388
*D* ^3^	493	649	0.17175	188.75000	−1.09384
*D* ^4^	37	37	0.01431	15.80000	−0.82874
*E* ^3^	376	281	0.17627	105.58333	−1.23426
*E* ^4^	18	18	0.01265	7.60000	−0.88654
*F* ^3^	368	270	0.16003	107.22222	−1.08462
*F* ^4^	23	23	0.01458	9.80000	−0.83399
*G* ^3^	484	384	0.17021	140.25000	−1.10811
*G* ^4^	19	19	0.00992	8.20000	−0.74443
*H* ^3^	476	373	0.15854	141.88889	−0.99021
*H* ^4^	24	24	0.01283	10.40000	−0.72278
*I* ^3^	2476	1911	0.18088	695.86111	−1.23455
*I* ^4^	105	105	0.01153	44.80000	−0.84470

^1^ Average pairwise nucleotide diversity per site. ^2^ Average pairwise nucleotide differences per sequence. Analysis performed on *Liberibacter* spp ^3^ or CLso ^4^. Concatenated sequences and nucleotides sites analysed: (A) *adk-mutS-icdA* (1037), (B) *adk-dnaG-icdA* (1012), (C) *adk-dnaG-recA* (1083)*,* (D) *adk-mutS-recA* (1108), (E) *adk-icdA* (602), (F) *adk-recA* (673), (G) *mutS-icdA* (830), (H) *mutS-recA* (901),and (I) *adk*-*dnaG*-*fumC*-*grpE*-*icdA*-*metG*-*mutS*, *purA*-*recA*-*gyrB* (3933).
